# Comment on prayer and healing: A medical and scientific perspective on randomized controlled trials

**DOI:** 10.4103/0019-5545.64587

**Published:** 2010

**Authors:** Abraham Verghese

**Affiliations:** Christian Medical College, Vellore, India. E-mail: averghese2002@yahoo.co.in

Sir,

I read with much interest the article "Prayer and Healing: A medical and scientific perspective on randomized control trials" by Dr. Chittaranjan Andrade and Rajiv Radhakrishnan.[1] They have made a critical analysis of the various research findings on the topic and report that they could not reach any conclusion. I am not surprised. They should not have expected a conclusive evidence in the first place. Prayer is an important postulate and practice in all religions. Science and religion are two important dimensions in one’s life; they are like the two sides of the same coin. Scientific claims are open to testing and verification. Religious claims cannot be proved and verified; they are to be experienced. Faith is the cornerstone of all religions. Any attempt to scientifically prove religious tenets will be a wild goose chase. Just because religious experiences cannot be proved as per the accepted scientific standards, it does not mean that they are not real or important. They are very important to the person who experiences them. That is why religion and spirituality are important in psychiatry.

When we try to understand our patients; we must explore their religious orientations and practices. This has been ignored all along. But, of late, many reports emphasize the importance of religion and spirituality in mental health, which I have elaborated in my paper "Spirituality and mental health."[2]
